# Global gene expression profiles of hematopoietic stem and progenitor cells from patients with chronic myeloid leukemia: the effect of in vitro culture with or without imatinib

**DOI:** 10.1002/cam4.1187

**Published:** 2017-10-13

**Authors:** Sócrates Avilés‐Vázquez, Antonieta Chávez‐González, Alfredo Hidalgo‐Miranda, Dafne Moreno‐Lorenzana, Lourdes Arriaga‐Pizano, Miguel Á. Sandoval‐Esquivel, Manuel Ayala‐Sánchez, Rafael Aguilar, Luis Alfaro‐Ruiz, Hector Mayani

**Affiliations:** ^1^ Oncology Research Unit Oncology Hospital National Medical Center Mexican Institute for Social Security Mexico City Mexico; ^2^ National Institute of Genomic Medicine Mexico City Mexico; ^3^ Immunochemistry Research Unit National Medical Center Mexican Institute for Social Security Mexico City Mexico; ^4^ Department of Hematology La Raza Medical Center Mexican Institute for Social Security Mexico City Mexico; ^5^ Department of Hip Surgery Villa Coapa General Hospital Mexican Institute for Social Security Mexico City Mexico

**Keywords:** Cell Biomarkers, chronic myeloid leukemia, gene expression profiles, hematopoietic stem/progenitor cells, Imatinib

## Abstract

In this study, we determined the gene expression profiles of bone marrow‐derived cell fractions, obtained from normal subjects and Chronic Myeloid Leukemia (CML) patients, that were highly enriched for hematopoietic stem (HSCs) and progenitor (HPCs) cells. Our results indicate that the profiles of CML HSCs and HPCs were closer to that of normal progenitors, whereas normal HSCs showed the most different expression profile of all. We found that the expression profiles of HSCs and HPCs from CML marrow were closer to each other than those of HSCs and HPCs from normal marrow. The major biologic processes dysregulated in CML cells included DNA repair, cell cycle, chromosome condensation, cell adhesion, and the immune response. We also determined the genomic changes in both normal and CML progenitor cells under culture conditions, and found that several genes involved in cell cycle, steroid biosynthesis, and chromosome segregation were upregulated, whereas genes involved in transcription regulation and apoptosis were downregulated. Interestingly, these changes were the same, regardless of the addition of Imatinib (IM) to the culture. Finally, we identified three genes—PIEZO2, RXFP1, and MAMDC2‐ that are preferentially expressed by CML primitive cells and that encode for cell membrane proteins; thus, they could be used as biomarkers for CML stem cells.

## Introduction

Chronic myeloid leukemia (CML) is a neoplastic hematological disease characterized by the abnormal overproduction and accumulation, both in blood and bone marrow, of myeloid cells. Its annual incidence is about 1.3 per 100,000 population, and it is slightly more common in males than in females 1. The Philadelphia chromosome, a truncated chromosome 22 resulting from the reciprocal translocation between DNA segments from chromosomes 9 and 22, is observed in more than 95% of CML patients; thus, it is recognized as the hallmark of the disease 2, 3. Such a translocation fuses the breakpoint cluster region (BCR) gene on chromosome 22 to the ABL gene on chromosome 9. The resultant chimeric gene—bcr‐abl oncogene encodes for a constitutively active tyrosine kinase protein that is central to the pathogenesis of CML, since it alters proliferation, cell death, and adherence/migration of the neoplastic cells 4.

In trying to find efficient drugs against CML, several tyrosine kinase inhibitors (TKIs) have been developed that specifically inhibit the action of the BCR‐ABL protein 5. Among them, Imatinib (IM) was the first to be developed and FDA‐approved, and today it has become a first‐line treatment worldwide 6, since major cytogenetic and molecular responses are achieved in the majority of patients that are treated with such a TKI 7, 8. It is noteworthy, however, that resistance to IM is observed in a proportion of patients, which can be attributed to different mechanisms, including point mutations in the ATP‐binding domain 9, 10, 11, 12.

Work by several laboratories has demonstrated that CML is a clonal disease that arises on a stem cell that, although abnormal in functional terms, shares several biological features with its normal counterpart. Indeed, both normal and CML hematopoietic stem cells (HSCs) are comprised within a cell population expressing the CD34 antigen, and lacking the expression of CD38 and any lineage‐specific antigen; so that they are referred to as lineage‐negative (Lin^−^) cells. The vast majority of normal and leukemic HSCs are quiescent, thus, leukemic HSCs (leukemic stem cells or LSCs) are insensitive to most chemotherapeutic agents, including tyrosine kinase inhibitors, such as IM 12, 13, 14, 15. Furthermore, both normal HSCs and LSCs reside in specific niches within the bone marrow; such niches play important roles in stem cell function and recent evidence suggests that they contribute to leukemic cell resistance to drugs by protecting them from the action of antineoplastic agents 16, 17.

Considering the above, finding specific differences between normal HSCs and LSCs has become a primary goal for several research groups, including ours. Some studies have shown that molecules such as CD25, CD26, IL1RAP, TPO‐R, and SIRT1 could be markers for LSCs 18, 19, 20, 21, 22; however, it is clear that further efforts are needed for a more complete characterization of LSCs. One relatively recent approach has been to assess global gene expression profiles of normal HSCs and LSCs, in order to identify particular genes that are differentially expressed in both cell types 23, 24, 25.

In trying to contribute to our understanding of the gene expression dynamics of HSCs in normal and leukemic conditions, in this study we performed a comparative analysis of the global gene expression profiles between LSCs from CML patients and normal HSCs. Our goal was to identify key genes and pathways—preferentially, or solely, expressed by LSCs—that could be used as markers for the identification and selection of LSCs (CD34^+^ CD38^−^ Lin^−^ cells), and as targets for inhibiting the growth of such cells. We also analyzed the population of progenitor cells (HPCs; CD34^+^ CD38^+^ Lin^−^ cells) since it has been clearly shown that these cells play an important role in the pathophysiology of CML 14. Finally, we asked whether in vitro culture of such cell populations, in the absence or presence of IM, could also result in significant changes in their gene expression profiles.

## Materials and Methods

### Patient samples

Bone marrow samples from five chronic‐phase CML patients at diagnosis were obtained at the Hematology Department, Medical Specialties Hospital, La Raza Medical Center, IMSS, Mexico City. Cytogenetic analysis was performed in all five patients at the time of diagnosis; all of them showed the Philadelphia chromosome in 100% of the cells analyzed (20–25 metaphases per patient). Bone marrow samples from five hematologically normal patients undergoing orthopedic surgery were obtained at the Hip Surgery Department, Villa Coapa Regional Hospital, IMSS, Mexico City. All samples were obtained after informed consent, according to the Ethics Committee of the IMSS National Medical Center.

### Isolation and culture of primitive cells

Mononuclear cells were isolated from bone marrow samples using Ficoll Paque Plus (GE Healthcare Life Sciences)‐based gradients. CD34^+^lin^−^ cells were enriched using the StemSep Human Hematopoietic Progenitor Cell Enrichment Kit (Stem Cell Technologies, Vancouver, Canada), according to the manufacturer's instructions. The enriched cells were collected in StemSpan medium, stained with CD34‐FITC and CD38‐APC antibodies (eBioscience, San Diego, CA), and sorted using a BD FACSAria (BD Biosciences, San Jose, CA) into hematopoietic progenitor (CD34^+^ CD38^+^Lin^−^; HPCs) and stem (CD34^+^ CD38^−^Lin^−^; HSCs) cells (Fig. S1). HSCs were immediately placed in Trizol and stored at −80°C until RNA extraction. HPCs were divided into three equal fractions. The first one was placed in Trizol and stored at −80°C for RNA extraction. The second one was cultured for 48 h in StemSpan Serum‐Free Expansion Media (Stem Cell Technologies, Vancouver, Canada) supplemented with the following cytokines: Erythropoietin (EPO); Thrombopoietin (TPO); Flt‐3‐ligand (Flt‐3L); Stem Cell Factor (SCF); Interleukin‐6 (IL6); IL3; Granulocyte Colony‐stimulating Factor (G‐CSF); and Granulocyte‐Macrophage CSF (GM‐CSF), each one at 10 ng/mL). The third fraction was cultured under the same conditions plus 2.5 *μ*mol/L IM (Gleevec, Novartis Pharmaceuticals). After culture, cells were washed and placed in Trizol.

### RNA extraction

Total RNA was extracted with Trizol (Invitrogen, Carlsbad, CA) according to the manufacturer's instructions. A NanoDrop Spectrophotometer ND‐1000 (Nanodrop Technologies, Wilmington, DE) was used to quantify the RNA extracts. The quality of extracted RNA was assessed using a Bioanalyzer 2100 (Agilent Technologies, Waldbronn, Germany) and samples with a RIN >7 were selected for performing microarray screening and analysis.

### Gene expression microarrays

One hundred and fifty nanogram of each RNA sample were processed using Affymetrix GeneChip Whole Transcript (WT) PLUS Reagent Kit (Affymetrix, Santa Clara, CA, USA). Fifteen microgram of cRNA was input into the second cycle cDNA reaction. 5.5 *μ*g of ss‐cDNA were input for fragmentation. Each DNA fragment was end labeled with biotin using terminal deoxynucleotidyl transferase before being hybridized to the arrays. Hybridization cocktails were prepared and applied to Gene Chip Human Gene 1.0 ST arrays (Affymetrix, Santa Clara, CA). Hybridization was performed at 60 rpm for 16 h at 45°C. Wash and stain processes were performed with the Genechip Expression Wash and Stain Kit in the GeneChip Fluidics Station 450 (Affymetrix, Santa Clara, CA). The probe arrays were scanned using the GeneChip Scanner 3000 7G and Affymetrix GeneChip Command Console Software (AGCC) (Affymetrix, Santa Clara CA, USA) to produce.CEL intensity files.

### Microarray analysis

Affymetrix Expression Console was used to process the original.CEL files. The.chp files were generated using the RMA‐sketch workflow after signal summarization (Median polish) and data normalization (Sketch‐Quantile method). Gene level analysis was further conducted with Affymetrix Transcriptome Analysis Console software. Twenty nine thousand and ninety‐six genes were tested at core level to compare their expression between four groups of chronic myeloid leukemia and hematologically normal controls: stem cells T0, progenitor cells T0, progenitor cells CTRL (cultured for 48 hrs without imatinib), progenitor cells IM (cultured for 48 h with 2.5 *μ*mol/L Imatinib). Genes with a fold change ≥2 or ≤ ‐2 and with a *P*‐value ≤0.05 were considered differentially expressed in each comparison. The Database for Annotation, Visualization, and Integrated Discovery (DAVID) tool (http://david.abcc.ncifcrf.gov/) was used to identify biological processes containing differentially expressed genes, according with the gene‐ontology classification. The microarray data are available at Gene Expression Omnibus (GEO; access number GSE97562).

### Real Time RT‐PCR

To validate the microarray results, cDNA was generated using M‐MLV Reverse Transcriptase and random primers (Invitrogen, Carlsbad, CA.) from RNA of cells from five different CML patients and five hematologically normal, hip‐replacement surgery adult patients (samples were different from the ones used for gene microarray). Five genes were selected for qPCR validation using gene‐specific TaqMan® assays (Life Technologies, Carlsbad, CA): PIEZO2 (Hs00401026_m1), MAMDC2 (Hs00299196_m1), RXFP1 (Hs01073141_m1), CDH2 (Hs00983056_m1), and ABI3BP (Hs00227206_m1). GAPDH (Hs03929097_g1) served as control for relative quantification. Quantitative PCR experiments were performed in a 7900 FAST real time thermal cycler (Applied Biosystems), the conditions used were: 95°C for 10 min, 40 cycles of 95°C for 10 sec and 60°C for 1 min; all samples were analyzed in triplicate. Fold change in gene expression was determined with the 2^−ΔΔCT^ method using GAPDH as the housekeeping gene.

## Results

### Gene expression profiles of CML HSCs are more similar to those of leukemic or normal HPCs than to the ones of normal marrow HSCs

**Figure 1:**
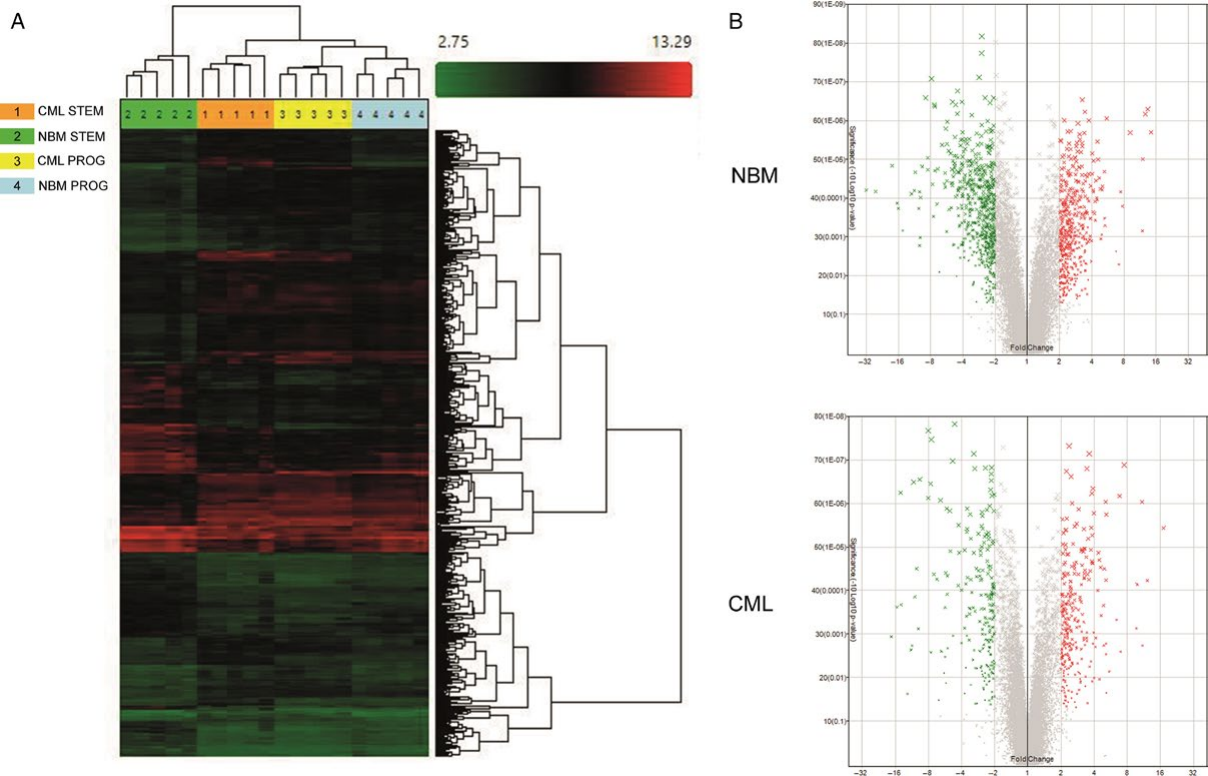
Differentially expressed genes between hematopoietic stem and progenitor cells from CML and normal bone marrow (NBM). (A) Hierarchical cluster shows 2298 genes differentially expressed between NBM and CML HSCs (STEM) and HPCs (PROG). Samples are in columns, genes are in rows. NBM‐ and CML‐derived CD34^+^ CD38^−^ Lin^−^ and CD34^+^ CD38^+^ Lin^−^ cells (*n* = 5) were compared using the TAC Affymetrix software, considering a fold change in 2.0 and *P* < 0.05. (B) The volcano plot shows 1093 genes differentially expressed between stem and progenitor cells from NBM (476 genes upregulated ‐red‐ and 617 downregulated ‐green‐ in progenitor cells), and 497 genes differentially expressed in CML stem and progenitor cells (280 genes upregulated ‐red‐ and 217 genes downregulated ‐green‐ in progenitor cells). The analysis was made using TAC Affymetrix software, considering a fold change in 2.0 and *P* < 0.05.

As a first approach, we performed a global comparison of the gene expression profiles from the four cell populations analyzed, that is, CD34^+^ CD38^−^Lin^−^ (enriched for HSCs) and CD34^+^ CD38^+^Lin^−^ (enriched for HPCs) cells from both, hematologically normal subjects and CML patients. By using Affymetrix Human Gene 1.0 ST array we assessed the expression of 29,096 genes and found the differential expression of 2298 genes between the four hematopoietic populations (ANOVA analysis; considering *P* < 0.05 and a fold change equal or higher than two, as a cut‐off). Hierarchical clustering showed that CML LSCs are transcriptionally more similar to CML progenitors than to their normal counterparts. Interestingly, the gene expression profiles of CML stem and progenitor cells were closer to the one of normal progenitors, whereas normal HSCs showed the most different expression profile of the four cell populations (Fig. 1A).

When we compared the gene expression profiles between stem and progenitor cells from normal bone marrow samples, we found 1093 genes differentially expressed (Fig. 1B). Of these, 476 were upregulated in HPCs (including ABI3BP, PREX2, HLF, EMP1, ARG2, DLK1, and CDH7) and 617 were downregulated (including CLC, MPO, CA1, CTSG, CD36, IRF8, CPA3, and ELANE). A similar comparison analysis between CML LSCs and HPCs showed 497 genes differentially expressed (Fig. 1C). Of these, 280 were upregulated in the progenitor population (including GAS2, PREX2, HLF, IGJ, AMICA1, CACNA1D, and HLA‐DQA1), whereas 217 genes were downregulated (including CLC, MPO, CA1, DH1, HDC, CD40L, LEF1, and MTSS1).

### CML LSCs overexpress genes involved in DNA repair, cell cycle, and chromosome condensation

**Figure 2:**
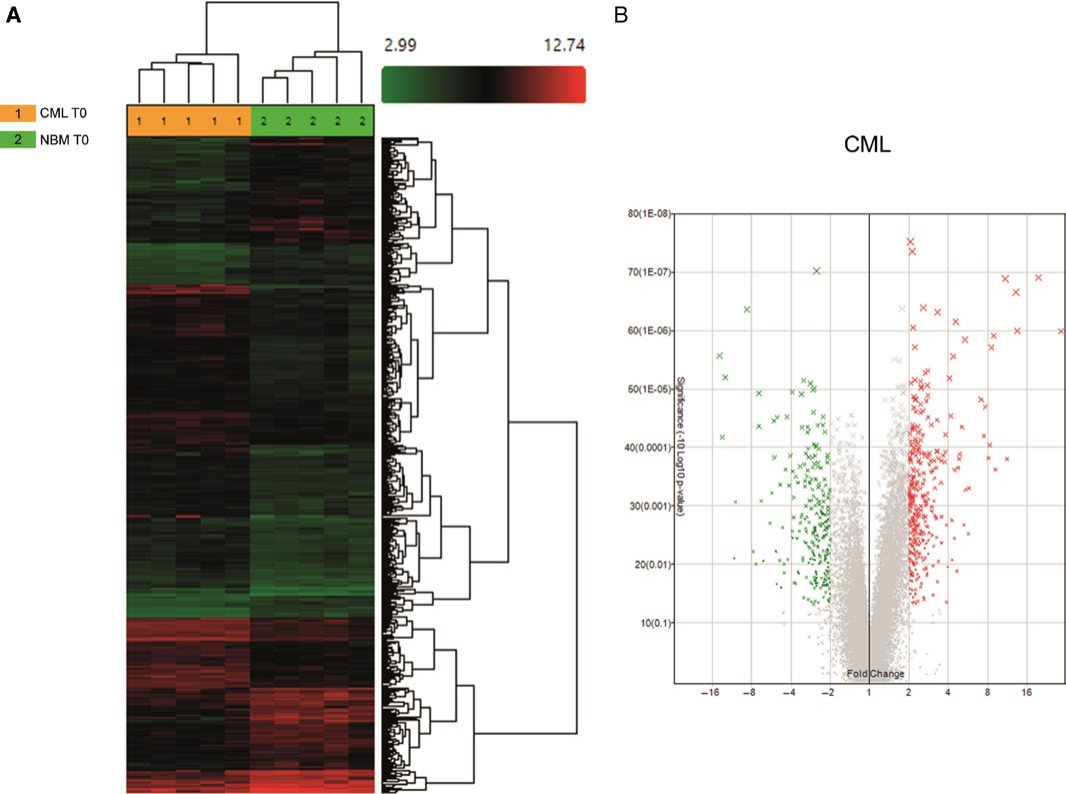
Differentially expressed genes between HSCs from CML and normal marrow. (A) Hierarchical clustering analysis using 584 differentially expressed genes between NBM and CML HSCs. Samples are in columns, genes are in rows. The analysis was made using TAC Affymetrix software, considering a fold change in 2.0 and *P* < 0.05. (B) Volcano plot of the 584 differentially expressed genes; 340 genes were upregulated (red) and 244 were downregulated (green) in CML cells. The analysis was made using TAC Affymetrix software considering 2.0 as a fold change and a *P* < 0.05.

**Figure 3:**
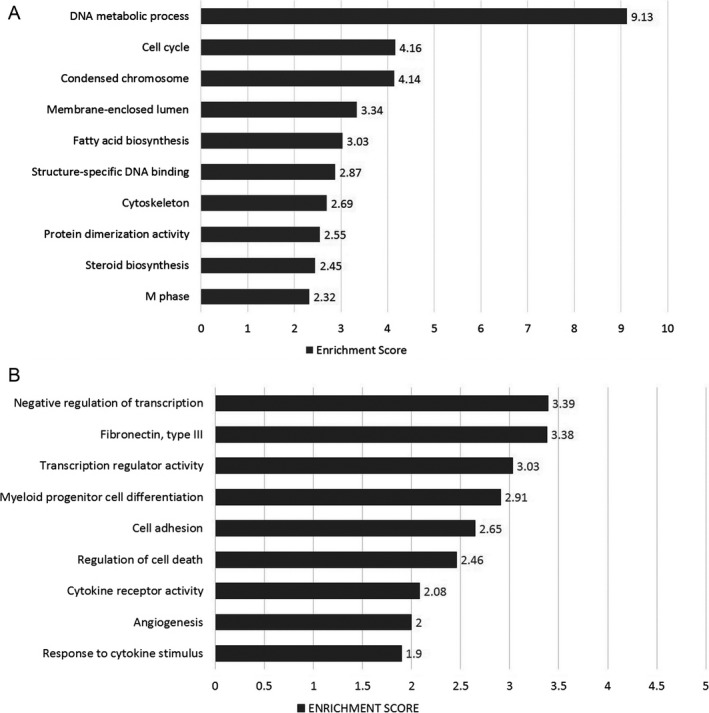
Most highly represented biologic processes/pathways that were upregulated (A) and downregulated (B) in CML HSCs as compared to their normal counterparts. Functional annotation in Gene Ontology categories was performed using the Database for Annotation, Visualization, and Integrated Discovery (DAVID) tool (http://david.abcc.ncifcrf.gov/) and were classified in Gene Ontology categories.

Comparative analysis of CD34^+^ CD38^−^Lin^−^ cells from CML and hematologically normal subjects showed the differential expression of 584 genes (Fig. 2A). Of these, 340 were upregulated in CML cells (Fig. 2B) ‐including genes like GAS2, RXFP1, MAMDC2, PIEZO2, HPGDS, CPA3, DPP4, CACNA1D, (Table S1)‐ and 244 were downregulated (Fig. 2B), as compared to normal cells, including ABI3BP, EMP1, PLAG1, PCDH9, ETV3, SELP, IL12RB2, and ID1 (Table S1). A functional annotation using DAVID software was performed to identify the biological processes that were preferentially represented, according with Gene Ontology (GO). When considering only upregulated genes, the most represented pathway was the one involving DNA metabolic processes, including genes that participate in DNA repair and replication, such as HMGB2, XRCC2, BLM, CHEK1, TYMS, CDC45, POLE2, FANCI, FANCF, and POLQ. The second most represented biologic process was cell cycle, including genes like CDC7, CDC6, HAUS6, KIF11, KIF15, SGOL1, KNTC1, and TTK. Genes that participate in chromosome condensation, including XPO1, MAD2L1, SGOL1, KNTC1, CASC5, SKA3, CENPK, and MLF1IP, corresponded to the third most represented biologic process (Fig. 3A).

When analysis was performed based on downregulated genes, the most represented process was negative regulation of transcription, with genes such as KLF4, KLF10, KLF11, ARID5A, ZHX2, PRDM16, ID2, ID1, BCL3, and ETV3. The Fibronectin Type III pathway was the second most downregulated pathway, including genes such as IL12RB2, IGF1R, SNED1, IL6ST, KAL1, SDK2, PTPRS, MPL, and ABI3BP. The third most downregulated pathway was cell adhesion, with genes such as ICAM1, SELP, EMCN, SDK2, PTPRS, PCDH9, NINJ1, CDH2, and PCDH17, among others (Fig 3B).

### CML progenitor cells overexpress genes involved in cholesterol biosynthesis and metabolism, and in the immune response

**Figure 4:**
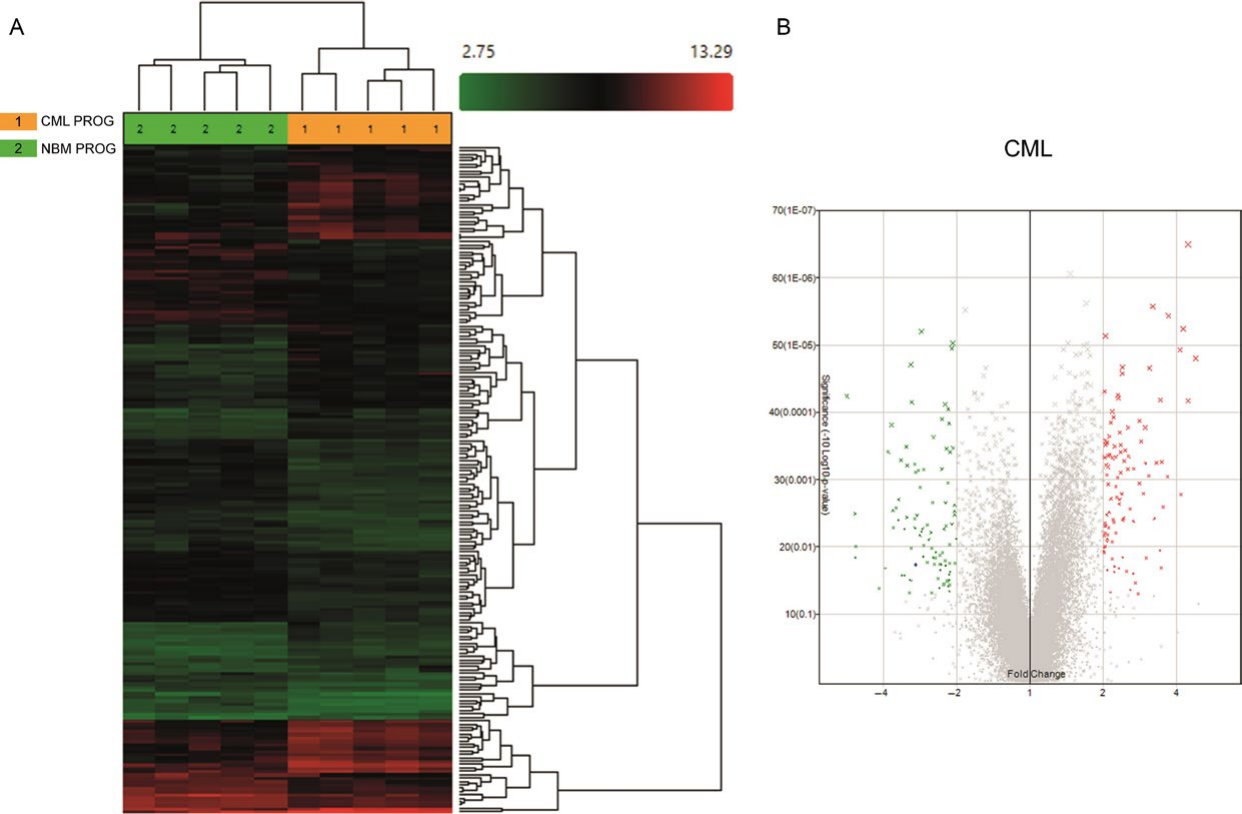
Differentially expressed genes between HPCs from CML and normal marrow. (A) Hierarchical clustering analysis using 198 differentially expressed genes between NBM and CML HSCs. Samples are in columns, genes are in rows. The analysis was made using TAC Affymetrix software, considering a fold change in 2.0 and *P* < 0.05. (B) Volcano plots of the 198 differentially expressed genes; 101 genes were upregulated (red) and 97 were downregulated (green) in CML cells. The analysis was made using TAC Affymetrix software considering 2.0 as a fold change and a *P* < 0.05.

**Figure 5:**
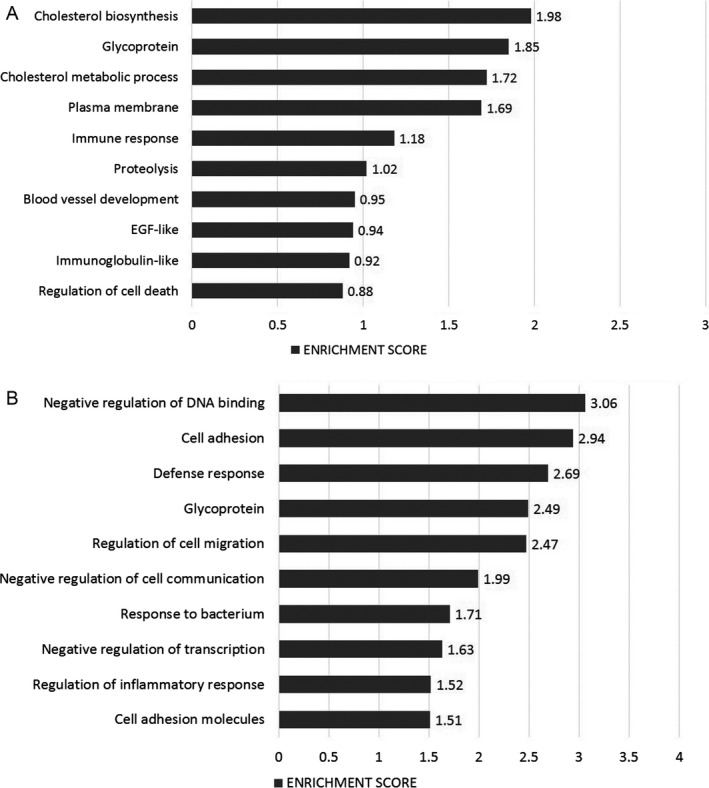
Most highly represented biologic processes/pathways that were upregulated (A) and downregulated (B) in CML HPCs as compared to their normal counterparts. Functional annotation in Gene Ontology categories was performed using the Database for Annotation, Visualization, and Integrated Discovery (DAVID) tool (http://david.abcc.ncifcrf.gov/) and were classified in Gene Ontology categories.

When comparing the gene expression profiles of CML and normal CD34^+^ CD38^+^Lin^−^ cells, we found the differential expression of 198 genes (Fig. 4A). One hundred and one genes were upregulated in CML cells (Fig. 4B), including KYNU, MSMO1, RXFP1, CFH, HDC, DHCR24, SCN9A, and CCL5 (Table S2), whereas 97 were downregulated (Fig. 4B), including IRF8, SKIL, KCNA3, ELANE, ARHGAP32, CRHBP, NBPF14, HCAR3, and ID2 (Table S2). When we performed the functional annotation, based on upregulated genes, the most represented biologic processes were cholesterol biosynthesis and metabolism, including genes such as HMGCR, CYP51A1, DHCR7, and DHCR24, glycoprotein biosynthesis, including GYPB, GYPE, GCNT2, MAMDC2, TSPAN2, MANSC1, KCNK5, and ANGPT2, and the immune response, with genes such as CFHR1, KYNU, ENPP2, CD40LG, IFITM3, NCF4, CFH, and CCL5 (Fig. 5A). In terms of downregulated genes, the most represented processes were regulation of DNA binding, including ICAM1, ID2, ID1, SMAD7, BCL3, NLRP3, and TRIB1, cell adhesion, including ICAM1, SELP, CDH9, PKP2, SELL, SDK2, NINJ1, CDH2, and CDH10, and defense response, including SELP, RNASE3, CXCL2, MNDA, LYZ, BCL3, NLRP3, CD180, BLNK, and CXCL10, among others (Fig. 5B).

### In vitro culture of normal and CML progenitor cells, with or without IM, induced changes in their gene expression profiles

In order to determine whether the in vitro culture of both normal and CML progenitor cells (CD34^+^ CD38^+^Lin^−^ cells) would result in any changes in their gene expression profiles, a fraction of them (200,000 cells/mL) was cultured, right after they were obtained by cell sorting. Cells were cultured for 48 h in serum‐free medium supplemented with 8 cytokines, with or without 2.5 *μ*mol/L IM. Cell numbers were also assessed to determine the effect of the drug on cell proliferation. As expected, cell proliferation was significantly inhibited by IM in cultures of CML progenitors (total cell numbers in the presence of IM were 52% of those at culture onset, before IM treatment); in contrast, in cultures of normal marrow‐derived cells, IM had no effect on the total cell number after 2 days of culture (Figure S2).

**Figure 6:**
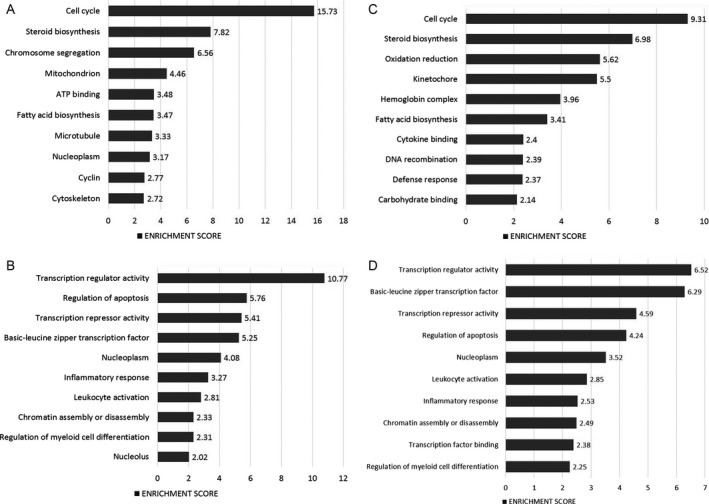
Most highly represented biologic processes/pathways in normal HPCs after in vitro culture. Figure shows the most upregulated (A) and downregulated (B) pathways after cells were cultured for 48 hours in the absence of IM, and the most upregulated (C) and downregulated (D) pathways when cells were cultured in the presence of the drug. Functional annotation in Gene Ontology categories was performed using the Database for Annotation, Visualization, and Integrated Discovery (DAVID) tool (http://david.abcc.ncifcrf.gov/) and were classified in Gene Ontology categories.

Evaluation of gene expression profiles was performed before and after the culture period. When normal marrow‐derived progenitors were cultured in the absence of IM, we found 1216 genes differentially expressed (489 upregulated and 727 downregulated) as compared to cells before culture. GO analysis based on upregulated genes showed that cell cycle, steroid biosynthesis and chromosome segregation were the most represented pathways (Fig. 6A), and genes such as MMP12, DHCR24, IL7R, CYP1B1, and GYPA were the most differentially expressed (Table S3). When considering downregulated genes, transcription regulation and apoptosis were the most represented biologic processes (Fig. 6B), and JUN, FOSB, AREG, FOS, and DUSP1 were the top genes downregulated (Table S3). When cells were cultured in the presence of IM, 928 genes were differentially expressed at the end of the culture period; 441 were upregulated and 487 downregulated. It is noteworthy that several of the pathways and genes most represented in cells cultured in the absence of IM were also represented in cells cultured in the presence of the drug (Fig. 6C,D; Table S4).

**Figure 7:**
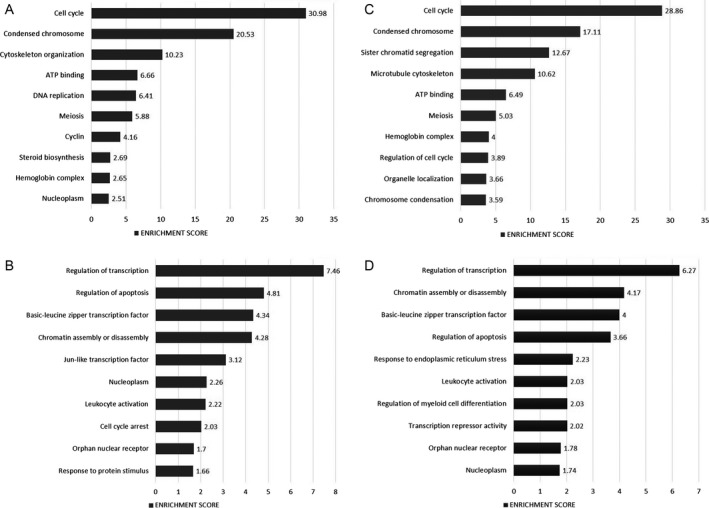
Most highly represented biologic processes/pathways in CML HPCs after in vitro culture. Figure shows the most upregulated (A) and downregulated (B) pathways after cells were cultured for 48 h in the absence of IM, and the most upregulated (C) and downregulated (D) pathways when cells were cultured in the presence of the drug. Functional annotation in Gene Ontology categories was performed using the Database for Annotation, Visualization, and Integrated Discovery (DAVID) tool (http://david.abcc.ncifcrf.gov/) and were classified in Gene Ontology categories.

When CML‐derived progenitors were cultured without IM, 498 genes were found to be upregulated and 375 were downregulated, for a total of 873 genes differentially expressed. Interestingly, biologic processes that were significantly represented in the GO analysis for normal cells were also observed in CML cells. That is to say, in terms of upregulated genes, such processes included cell cycle, chromosome condensation, steroid biosynthesis and ATP binding (Fig. 7A), whereas transcription regulation and apoptosis were among the most represented in terms of downregulated genes (Fig. 7B). Some of the most upregulated genes included GYPA, HBA2, CYP1B1, PLK1, and PSAT1. On the other hand, JUN, FOSB, FOS, NR4A2, and DUSP1 were among the most downregulated genes (Table S5). When CML cells were cultured with IM, 752 genes were differentially expressed; 455 genes were upregulated and 297 were downregulated. As for normal cells, several of the pathways and genes most represented in CML cells cultured in the absence of IM were also represented in cells cultured in its presence (Fig. 7C,D; Table S6).

### PIEZO2, RXFP1, and MAMDC2 are preferentially expressed in CML HSCs

**Figure 8:**
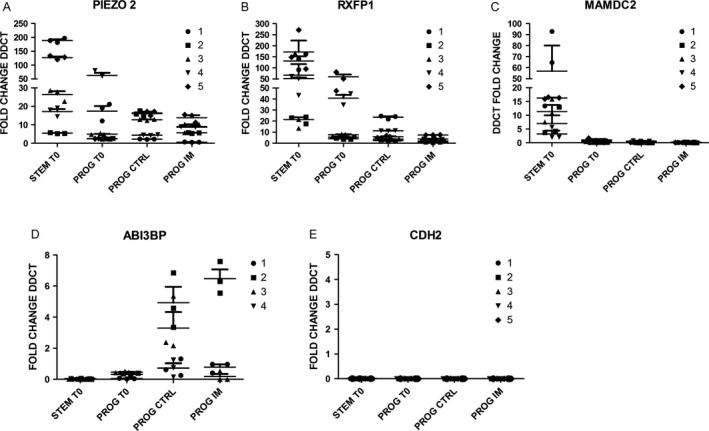
Quantification of five representative genes by real time qRT‐PCR. Among all the genes that were differentially expressed between CML and NBM cells, based on microarray data, PIEZO2, RXFP1, MAMDC1, CDH2, and ABI3BP were selected to be assessed by qRT‐PCR. Cell fractions analyzed corresponded to HSCs (STEM T0), HPCs before culture (PROG T0), HPCs after 48 hours of culture in the absence (PROG CTRL) or presence (PROG IM) of IM. The relative amount of each gene was determined using the ΔΔCt method, normalized to GAPDH and NBM. For each gene quantification, five cell samples were used (1–5), that were different from those used for microarray analysis.

Based on the profile of individual genes that showed high expression levels in CML LSCs, as compared to their levels in normal HSCs (see list in Table S1), we focused on three particular genes: PIEZO2, RXFP1, and MAMDC2; so, their expression was determined by quantitative real‐time PCR. Confirming the results shown in Table S1, we found that all three genes were markedly expressed in leukemic LSCs (Fig. 8). Interestingly, whereas PIEZO2 (Fig. 8A) and RXFP1 (Fig. 8B) were also expressed in leukemic HPCs at significant levels, MAMDC2 was practically absent in the progenitor population (Fig. 8C). When leukemic progenitors were cultured in the absence or in the presence of IM, PIEZO2 and, to a lesser extent RXFP1, were still expressed; in contrast, MAMDC2 expression was not detected. In terms of genes that were downregulated in CML cells, we focused on ABI3BP and CDH2 (Fig. 8D and E). Expression levels of the former were very low in both CML LSCs and HPCs. Interestingly such levels were significantly increased in both cell types after the culture period, both in the absence or in the presence of IM (Fig. 8D). CDH2 expression levels, in contrast, were undetectable in CML progenitors at any condition (Fig. 8E).

## Discussion

Among all human malignancies known to date, CML is arguably the best known in terms of its biology. Evidence presented by different groups indicate that this hematologic disorder arises at the level of a HSC that is transformed by means of a chromosomal translocation, resulting in the generation of a chimeric gene (*bcr‐abl*), which encodes for an abnormally active tyrosine kinase. Such a cytogenetic and molecular alteration has been recognized as the hallmark of the disease, and in vivo animal models have shown that *bcr‐abl* is the sole molecular change responsible for CML induction 26. Selective elimination of CML LSCs by targeted therapies has become a major goal in current hematology. Many efforts are being made in trying to develop novel and more effective ways, including both *bcr‐abl*‐dependent and independent mechanisms 27, 28, to kill CML LSCs without affecting their normal counterparts. Evidently, such an objective requires the full identification and physiological characterization of these primitive cells.

Normal and CML‐derived HSCs share several biological features, including their immunophenotype (CD34^+^ CD38^−^ Lin^−^), their quiescence, their capacity to self‐renew, and their ability to differentiate into myeloid, erythroid and lymphoid lineages. Indeed, in contrast to acute myeloid leukemia, in which there is a maturation arrest in the myeloid lineage, in CML the maturation program of the neoplastic cells does not seem to be altered, so that morphologically normal cells are being generated throughout the chronic phase of the disease 29. In functional terms, however, significant differences have been observed between normal HSCs and CML LSCs, both in vitro and in vivo 13, 30. Such differences result from alterations in the molecular biology of leukemic LSCs that impact on their physiology. Hematopoietic progenitors from CML also show an altered biology, as compared to their normal counterparts, both in vivo and in vitro 30.

During the last few years, there has been great interest in assessing global gene expression profiles of HSCs and LSCs in order to identify particular genes that are differentially expressed in each cell type 23, 24, 25. Genomic approaches have shown molecular signatures that distinguish chronic phase from accelerated phase and blast crisis at the MNC level 23, and at the level of CD34^+^ cells 31. In trying to contribute to our understanding of the molecular biology of CML stem and progenitor cells at the genomic level, in this study, we have performed a comparative analysis of the global gene expression profiles of CML and normal hematopoietic stem and progenitor cells. Using this approach, we have identified some of the major pathways and specific genes that are differentially expressed in CML cells, as compared to their normal counterparts. We have further analyzed the expression profiles of these cells after they have been cultured in vitro, with or without IM.

Except for the work by Gerber and colleagues 25, most studies reported to date on the gene expression profiles of CML cells have used whole MNC or CD34^+^ cells as the target population. Thus, one of our initial goals was to perform such studies in specific CD34^+^ cell subpopulations, so we could assess differences between HSCs and HPCs from both normal subjects and CML patients. By using a cell sorting approach, we were able to obtain practically pure cell populations in terms of their immunophenotype (CD34^+^ CD38^−^ Lin^−^ cells, enriched for HSCs, and CD34^+^ CD38^+^ Lin^−^ cells, enriched for HPCs). However, in the case of CML‐derived cells, such populations most likely consisted of a mixture of both Ph^+^ and Ph^−^ cells, since it is well known that in CML marrow, both leukemic and normal cells coexist within the same population. Although at this time we cannot rule out the possibility that the presence of residual normal cells within the CML cell fractions could have influenced the results obtained, it was clear that each one of the four cell populations analyzed showed a particular gene expression profile. This suggests that the profiles observed for the leukemic cells are true reflections of the gene expression patterns of CML LSCs and HPCs.

When we compared the gene expression profiles of the four cell populations obtained, we found that the profiles of CML stem and progenitor cells were closer to the one of normal progenitors, whereas normal HSCs showed the most different expression profile of all. We also found that the expression profiles of LSCs and HPCs from CML marrow were closer to each other than the ones of HSCs and HPCs from normal marrow. Although it has been clearly shown that the LSC pool in CML is heterogeneous—consisting of several cell stages that differ in their capacity to initiate and sustain the leukemic population 21‐ the exact cell of origin of leukemia and the mechanisms resulting in malignant transformation are still poorly understood. In this regard, our results seem to suggest that, in CML, malignant transformation occurs within the HSC pool in a cell stage that is closer to the progenitor cell compartment than to the long‐term HSC stage. Thus, the genomic profiles of LSCs are more similar to those of normal—and leukemic‐HPCs than to those of normal HSCs. Interestingly, and supporting this idea, Jamieson and colleagues have presented evidence indicating that in blast crisis CML, granulocyte‐macrophage progenitors may be the actual LSCs 32. It is noteworthy, however, that our observations are in contrast to those of Gerber et al. These authors used a similar approach to ours and found that global gene expression patterns between normal HSCs and CML LSC‐enriched fractions were closer to each other than normal HSCs were to their matched HPC‐enriched cell fraction 25. They also found that only 97 genes were differentially expressed between normal and CML CD34^+^ CD38^−^ Lin^−^ cells, which clearly differs from our study, in which 584 genes were differentially expressed. The reason(s) for such discrepancies are not clear; however, one possible explanation is that the cell populations analyzed in both studies were not exactly the same. That is to say, in their study, Gerber and colleagues included ALDH expression levels as part of their cell purification strategy. We did not include such a molecule; thus, it is possible that the different results observed could be due, at least in part, to the differences in the cell sorting strategies that, in turn, resulted in cell fractions that are not absolutely comparable.

In spite of these differences, it is noteworthy that in the study by Gerber et al. as well as in our own study, analysis of the expression profiles of CML LSCs showed overexpression of genes involved in DNA repair, cell cycle and chromosome condensation, whereas genes involved in myeloid cell differentiation were downregulated. At the individual gene level, both studies, Gerber's and ours, showed that GAS2 and DPP4 were among the most overexpressed genes in CML HSCs. The protein encoded by GAS2 is a Caspase‐3 substrate that plays important roles in cell shape, apoptosis, cell cycle, and calpain activities 33, 34. GAS2 expression is altered in different types of cancer, such as colorectal cancer 35, and it is important to note that this gene has been found to be overexpressed in CD34^+^ cells from CML patients 36. Similarly, DPP4 (CD26), a cell membrane enzyme that specifically disrupts the SDF1‐CXCR4 axis, has been found to be highly expressed in CML CD34^+^ CD38^−^ Lin^−^ cells, whereas its expression in normal HSCs is low/absent 19. This has led to the proposal that CD26 could be used as a biomarker for CML LSCs. Our results are clearly in keeping with those previous studies.

Among all the genes that were overexpressed in CML LSCs, we focused on three of them: PIEZO2, RXFP1 and MAMDC2. The reason to focus on those particular genes was a threefold. First, all of them showed a > 13‐fold‐change expression value, as compared to their expression levels in normal HSCs. Second, these three genes encode for cell membrane proteins, which indicates that such proteins could be used as potential biomarkers for CML LSCs. Third, although their function in some other cell types has been determined, their function in CML LSCs is not known. Indeed, it has been shown that PIEZO2 encodes for a transmembrane protein that has a role in mechanosensation, including rapidly adapting mechanically activated currents in somatosensory neurons 37, 38. It has been reported that the majority of the myelinated sensory neurons projecting to the bone marrow express PIEZO2 39, and that this gene plays an important role as a critical regulator of tumor angiogenesis and vascular permeability 40. Regarding the function of RXFP1, also known as LGR7, it is known that this gene encodes for a G protein‐coupled cell membrane protein that is the receptor for relaxin 41. Relaxin can exert a wide range of effects, including vasodilatation, anti‐fibrotic effects, angiogenic, anti‐apoptoptotic and anti‐inflammatory effects 42. Recent reports indicate that RXFP1 participates in the development of several types of cancer, including prostate cancer 43, 44. In contrast to these two proteins, the role of MAMDC2 is still not clear. Its paralog MDGA2/MAMDC1 binds to CAMs, participating in cell adhesion; thus, it is possible that MAMDC2 is also involved in cell adhesion mechanisms. MDGA2/MAMDC1 acts as an antitumor molecule in gastric cancer 45; however, the role of MAMDC2 in cancer, if any, has yet to be elucidated. Interestingly, MAMDC2 gene is mutated in patients with Kabuki syndrome, a multiple congenital anomaly/mental retardation syndrome characterized by a distinct facial appearance 46. To date, the actual function of these three proteins in LSCs are still not understood, and further studies are needed to determine their particular roles in the physiology of CML stem cells. Nonetheless, as mentioned above, these proteins could be used as biomarkers, with potential biological and clinical relevance, upon confirmation—in larger series of patients—of their high (selective) expression in LSCs from CML patients.

Regarding the expression profiles observed in hematopoietic progenitors, we found that gene pathways involved in cholesterol metabolism were among the most represented pathways. The significance of this observation is still not clear, however, it is noteworthy that a high incidence of low cholesterol values has been observed in CML patients 47, and some groups have found that such hypocholesterolemia can have prognostic significance 48. In terms of downregulated pathways, cell adhesion was one of the most highly represented. This is in keeping with the fact that cell adhesion mechanisms are disrupted in CML, leading to the mobilization of immature cells into circulation. Indeed, it has been clearly shown, both in vitro and in vivo, that cell adhesion is one of the major biological processes that are affected in CML cells 1, 2, 49.

Recent work by John Dick's group in AML patients has shown that rather than immunophenotype, gene expression signatures define LSC function 50. That is to say, it seems that stem cell expression programs are not exclusive of the CD34^+^ CD38^−^ Lin^−^ cell population, but persist in the progenitor (CD34^+^ CD38^+^ Lin^−^ cells) and blast (CD34^−^ CD38^+/−^ cells) populations, although at significantly lower levels. These and other observations point to the need for the development and validation of new LSC biomarkers. This is also true for stem cells in solid tumors. In keeping with this notion, this study contributes to the best of our knowledge on the global gene expression signatures of distinct primitive normal and leukemic hematopoietic cell populations, and identifies three new potential biomarkers (PIEZO2, RXFP1, and MAMDC2) for LSC.

Trying to understand, at the genomic level, the effects of IM and other tyrosine kinase inhibitors on CML patients, and the molecular nature of TKI resistance, based on gene expression profiles, has been an important goal for several groups. In this regard, significant progress has been made over the last few years 23, 24, 51, 52, 53. To date, however, and to the best of our knowledge, the genomic changes induced by in vitro administration of IM to pure populations of HPCs have not been reported. In trying to determine whether the in vitro culture of both normal and CML progenitor cells would affect their gene expression profiles, such cell populations were cultured for a two‐day period under standard conditions, without or with IM. It is important to point out that this type of experiments were not conducted in cultures of HSCs or LSCs due to the extremely low number of cells obtained.

In vitro culture of HPCs resulted in significant changes in their gene expression profiles. For normal cells, over 1200 genes were differentially expressed; cell cycle, steroid biosynthesis and chromosome segregation were among the most upregulated pathways, whereas transcription regulation and apoptosis were among the most downregulated biologic processes. When IM was added to the cultures we observed that the expression of almost one thousand genes was altered and several of the pathways and genes most represented in cells cultured in the absence of IM were also represented in cells cultured in the presence of the drug. Culture of CML progenitors also caused changes in the expression of several hundred genes (over 850 genes in cultures without IM and over 750 genes in the presence of the drug). As for normal cells, cell cycle, chromosome condensation, steroid biosynthesis and ATP binding were among the most represented biologic processes that were upregulated, whereas transcription regulation and apoptosis were among the most represented pathways in terms of downregulated genes. These findings indicate that the simple fact of culturing cells in serum‐free liquid medium in the presence of an eight‐cytokine combination induced important changes in the expression patterns of a significant number of genes. It seems very likely that such changes were induced by the stimulatory cytokines added to the culture medium; however, further studies should be performed to determine the actual factor(s) causing such changes. Interestingly, the changes observed were similar regardless of the absence or the presence of IM. The reason for this is not completely clear, however, it seems very likely that the eight‐cytokine cocktail exerted such a strong effect, “forcing” the cells to display a particular gene expression profile, and the presence of IM changed such a profile only in a slight manner. In CML cells, the reduced proliferation observed in cultures supplemented with IM could be due to changes in the expression of a particular gene (including one or more of those listed in Table S6). However, it is also possible that such an effect is not due to significant changes, but rather subtle changes in the level of expression of specific genes.

In summary, in this study we have determined the gene expression profiles of bone marrow‐derived cell fractions, obtained from normal subjects and CML patients, that were highly enriched for hematopoietic stem (HSCs) and progenitor (HPCs) cells. Our results indicate that the profiles of CML stem and progenitor cells were closer to the one of normal progenitors, whereas normal HSCs showed the most different expression profile of all. We found that the expression profiles of LSCs and HPCs from CML marrow were closer to each other than the ones of HSCs and HPCs from normal marrow. The major biologic processes dysregulated in CML cells included DNA repair, cell cycle, chromosome condensation, cell adhesion, and the immune response. We have also determined the genomic changes in both normal and CML progenitor cells under culture conditions, and found that several genes involved in cell cycle, steroid biosynthesis and chromosome segregation were upregulated, whereas genes involved in transcription regulation and apoptosis were downregulated. Interestingly, these changes were basically the same, regardless of the addition of IM to the culture. Finally, we have identified three genes—PIEZO2, RXFP1, and MAMDC2‐ that are preferentially expressed by CML primitive cells and that encode for cell membrane proteins; thus, they are potential biomarkers for CML stem cells.

## Conflict of Interest

None declared.

## Supporting information


**Table S1.** Top ten genes differentially expressed between normal and CML HSCs.
**Table S2.** Top ten genes differentially expressed between normal and CML HPCs.
**Table S3**: Top ten genes differentially expressed between normal HPCs before and after in vitro culture without IM.
**Table S4.** Top ten genes differentially expressed between normal HPCs before and after in vitro culture with IM.
**Table S5.** Top ten genes differentially expressed between CML HPCs before and after in vitro culture without IM.
**Table S6.** Top ten genes differentially expressed between CML HPCs before and after in vitro culture with IM.
**Figure S1.** Purification of HSCs and HPCs.
**Figure S2.** Effect of IM on the proliferation of HPCs derived from normal (NBM) and CML bone marrow.
