# Supplementary material for: Global gene expression profiles of hematopoietic stem and progenitor cells from patients with chronic myeloid leukemia: the effect of in vitro culture with or without imatinib
Source: Cancer Med. 2017 Oct 13;6(12):2942–56. doi: 10.1002/cam4.1187 (PMC5727298; doi:10.1002/cam4.1187)
Supplement: Supplementary file 1 — Table S1. Top ten genes differentially expressed between normal and CML HSCs. Table S2. Top ten genes differentially expressed between normal and CML HPCs. Table S3: Top ten genes differentially expressed between normal HPCs before and after in vitro culture without IM. Table S4. Top ten genes differentially expressed between normal HPCs before and after in vitro culture with IM. Table S5. Top ten genes differentially expressed between CML HPCs before and after in vitro culture without IM. Table S6. Top ten genes differentially expressed between CML HPCs before and after in vitro culture with IM. Figure S1. Purification of HSCs and HPCs. Figure S2. Effect of IM on the proliferation of HPCs derived from normal (NBM) and CML bone marrow. [file CAM4-6-2942-s001.docx]

**TABLE S1**

**TOP TEN GENES DIFFERENTIALLY EXPRESSED BETWEEN NORMAL AND CML HSCs**

| Gene Symbol | Gene name | Fold change | p-Value | FDR |
| --- | --- | --- | --- | --- |
| GAS2 | growth arrest-specific 2 | **29.11** | 0.000001 | 0.002113 |
| RXFP1 | relaxin/insulin-like family peptide receptor 1 | **19.5** | 1.24E-07 | 0.000758 |
| MAMDC2 | MAM domain containing 2 | **13.39** | 9.97E-07 | 0.002113 |
| PIEZO2 | piezo-type mechanosensitive ion channel component 2 | **13.15** | 2.22E-07 | 0.001076 |
| HPGDS | hematopoietic prostaglandin D synthase | **11.16** | 0.000157 | 0.020181 |
| CPA3 | carboxypeptidase A3 (mast cell) | **9.17** | 0.00024 | 0.023154 |
| DPP4 | dipeptidyl-peptidase 4 | **8.52** | 0.000002 | 0.003124 |
| PTPRD | protein tyrosine phosphatase, receptor type, D | **8.28** | 0.000091 | 0.017957 |
| CACNA1D | calcium channel, voltage-dependent, L type, alpha 1D subunit | **8.05** | 0.000151 | 0.02005 |
| MSMO1 | methylsterol monooxygenase 1 | **7.66** | 0.00002 | 0.010612 |
| ETV3 | ets variant 3 | **-7.03** | 0.000044 | 0.013483 |
| PCDH9 | protocadherin 9 | **-7.05** | 0.000012 | 0.008027 |
| NR4A1 | nuclear receptor subfamily 4, group A, member 1 | **-7.39** | 0.00985 | 0.110612 |
| METTL24 | methyltransferase like 24 | **-7.77** | 0.006062 | 0.088629 |
| PLAG1 | pleiomorphic adenoma gene 1 | **-8.69** | 4.34E-07 | 0.001403 |
| SKIL | SKI-like oncogene | **-10.62** | 0.000869 | 0.037752 |
| FIGN | Fidgetin | **-10.91** | 0.00784 | 0.099114 |
| FAM19A2 | family with sequence similarity 19 (chemokine (C-C motif)-like), member A2 | **-12.68** | 0.000006 | 0.007243 |
| EMP1 | epithelial membrane protein 1 | **-13.37** | 0.000066 | 0.015424 |
| ABI3BP | ABI family, member 3 (NESH) binding protein | **-14.06** | 0.000003 | 0.004017 |

Top ten genes upregulated and downregulated between CML and NBM HSCs, according to the analysis performed with Affymetrix transcriptome analysis console. Fold Change > 2; p <0.05.

**TABLE S2**

**TOP TEN GENES DIFFERENTIALLY EXPRESSED BETWEEN NORMAL AND CML HPCs**

| Gene Symbol | Gene name | Fold change | p-Value | FDR |
| --- | --- | --- | --- | --- |
| KYNU | kynureninase (L-kynurenine hydrolase) | **4.78** | 0.000016 | 0.022009 |
| MSMO1 | methylsterol monooxygenase 1 | **4.45** | 0.000068 | 0.036407 |
| RXFP1 | relaxin/insulin-like family peptide receptor 1 | **4.45** | 3.20E-07 | 0.009322 |
| CFH | complement factor H | **4.24** | 0.000006 | 0.020357 |
| HDC | histidine decarboxylase | **4.17** | 0.001673 | 0.091594 |
| DHCR24 | 24-dehydrocholesterol reductase | **4.13** | 0.000012 | 0.020357 |
| SCN9A | sodium channel, voltage-gated, type IX, alpha subunit | **3.69** | 0.000004 | 0.017817 |
| CCL5 | chemokine (C-C motif) ligand 5 | **3.51** | 0.002565 | 0.103662 |
| PIEZO2 | piezo-type mechanosensitive ion channel component 2 | **3.46** | 0.000546 | 0.061798 |
| ANGPT2 | angiopoietin 2 | **3.43** | 0.000065 | 0.036407 |
| ZFP36 | ZFP36 ring finger protein | **-3.69** | 0.000154 | 0.045739 |
| ID2 | inhibitor of DNA binding 2, dominant negative helix-loop-helix protein | **-3.82** | 0.000387 | 0.059006 |
| HCAR3 | hydroxycarboxylic acid receptor 3 | **-3.92** | 0.020908 | 0.212205 |
| NBPF14 | neuroblastoma breakpoint family, member 14 | **-4.15** | 0.041335 | 0.258699 |
| CRHBP | corticotropin releasing hormone binding protein | **-5.18** | 0.009848 | 0.160345 |
| ARHGAP32 | Rho GTPase activating protein 32 | **-5.2** | 0.014604 | 0.189113 |
| ELANE | elastase, neutrophil expressed | **-5.22** | 0.003229 | 0.113648 |
| KCNA3 | potassium voltage-gated channel, shaker-related subfamily, member 3 | **-5.65** | 0.000057 | 0.036407 |
| SKIL | SKI-like oncogene | **-6.96** | 0.00096 | 0.073001 |
| IRF8 | interferon regulatory factor 8 | **-7.04** | 0.002576 | 0.103682 |

Top ten genes upregulated and downregulated between CML and NBM HPCs, according to the analysis performed with Affymetrix transcriptome analysis console. Fold Change > 2; p <0.05.

**TABLE S3**

**TOP TEN GENES DIFFERENTIALLY EXPRESSED BETWEEN NORMAL HPCs BEFORE AND AFTER IN VITRO CULTURE WITHOUT IM**

| Gene Symbol | Gene name | Fold change | p-Value | FDR |
| --- | --- | --- | --- | --- |
| MMP12 | matrix metallopeptidase 12 | **28.57** | 0.000491 | 0.006669 |
| DHCR24 | 24-dehydrocholesterol reductase | **19.47** | 4.23E-08 | 0.000103 |
| IL7R | interleukin 7 receptor | **15.15** | 0.000111 | 0.002865 |
| CYP1B1 | cytochrome P450, family 1, subfamily B, polypeptide 1 | **14.39** | 0.000002 | 0.000535 |
| GYPA | glycophorin A (MNS blood group) | **13.91** | 0.000078 | 0.002359 |
| MRC1 | mannose receptor, C type 1 | **13.31** | 0.000638 | 0.007906 |
| HBG1; HBG2 | hemoglobin, gamma A; hemoglobin, gamma G | **10.89** | 7.52E-08 | 0.000103 |
| MSMO1 | methylsterol monooxygenase 1 | **10.79** | 1.57E-07 | 0.000148 |
| S100A8 | S100 calcium binding protein A8 | **10.53** | 0.005526 | 0.033125 |
| CCL2 | chemokine (C-C motif) ligand 2 | **8.84** | 0.0001 | 0.002704 |
| KLF4 | Kruppel-like factor 4 | **-13.56** | 0.000012 | 0.001006 |
| NR4A2 | nuclear receptor subfamily 4, group A, member 2 | **-14.63** | 0.000002 | 0.000515 |
| NR4A1 | nuclear receptor subfamily 4, group A, member 1 | **-15.84** | 3.26E-07 | 0.000207 |
| RGS1 | regulator of G-protein signaling 1 | **-15.93** | 0.000004 | 0.000674 |
| TNFAIP3 | tumor necrosis factor, alpha-induced protein 3 | **-17.39** | 0.000009 | 0.000847 |
| DUSP1 | dual specificity phosphatase 1 | **-19.88** | 5.03E-08 | 0.000103 |
| FOS | FBJ murine osteosarcoma viral oncogene homolog | **-20.12** | 0.000011 | 0.000961 |
| AREG | amphiregulin | **-34.22** | 0.000258 | 0.004609 |
| FOSB | FBJ murine osteosarcoma viral oncogene homolog B | **-36.01** | 1.86E-07 | 0.000159 |
| JUN | jun proto-oncogene | **-62.62** | 8.58E-08 | 0.000104 |

Top ten genes upregulated and downregulated between NBM HPCs cultured without IM for 48 hrs vs HPCs at time 0 (before culture), according to the analysis performed with Affymetrix transcriptome analysis console. Fold Change > 2.0; p <0.05.

**TABLE S4**

**TOP TEN GENES DIFFERENTIALLY EXPRESSED BETWEEN NORMAL HPCs BEFORE AND AFTER IN VITRO CULTURE WITH IM**

| Gene Symbol | Gene name | Fold change | p-Value | FDR |
| --- | --- | --- | --- | --- |
| MMP12 | matrix metallopeptidase 12 | **25.5** | 0.000243 | 0.006535 |
| DHCR24 | 24-dehydrocholesterol reductase | **19.31** | 2.01E-08 | 0.000065 |
| IL7R | interleukin 7 receptor | **17.33** | 0.000465 | 0.009341 |
| CYP1B1 | cytochrome P450, family 1, subfamily B, polypeptide 1 | **16.89** | 2.82E-07 | 0.000256 |
| HBG1 | hemoglobin, gamma A; hemoglobin, gamma G | **14.74** | 1.64E-08 | 0.000065 |
| GYPA | glycophorin A (MNS blood group) | **14.28** | 0.000015 | 0.001585 |
| MRC1 | mannose receptor, C type 1 | **13.38** | 0.000851 | 0.013032 |
| S100A8 | S100 calcium binding protein A8 | **13.08** | 0.007049 | 0.049329 |
| MSMO1 | methylsterol monooxygenase 1 | **12.12** | 1.61E-07 | 0.000203 |
| GYPB | glycophorin B (MNS blood group) | **9.77** | 0.000039 | 0.002496 |
| KLF4 | Kruppel-like factor 4 (gut) | **-13.98** | 0.00001 | 0.001335 |
| NR4A2 | nuclear receptor subfamily 4, group A, member 2 | **-14.16** | 0.000002 | 0.00057 |
| ZFP36 | ZFP36 ring finger protein | **-14.31** | 4.54E-07 | 0.000266 |
| RGS1 | regulator of G-protein signaling 1 | **-15.75** | 0.000005 | 0.000982 |
| TNFAIP3 | tumor necrosis factor, alpha-induced protein 3 | **-16.85** | 0.000005 | 0.000982 |
| FOS | FBJ murine osteosarcoma viral oncogene homolog | **-17.97** | 0.00001 | 0.001335 |
| DUSP1 | dual specificity phosphatase 1 | **-20.64** | 1.12E-08 | 0.000065 |
| FOSB | FBJ murine osteosarcoma viral oncogene homolog B | **-26.17** | 2.27E-08 | 0.000066 |
| AREG | Amphiregulin | **-41.61** | 0.000228 | 0.006325 |
| JUN | jun proto-oncogene | **-48.37** | 9.53E-07 | 0.000391 |

Top ten genes upregulated and downregulated between NBM HPCs cultured with IM for 48 hrs vs HPCs at time 0 (before culture), according to the analysis performed with Affymetrix transcriptome analysis console. Fold Change > 2.0; p <0.05

**TABLE S5**

**TOP TEN GENES DIFFERENTIALLY EXPRESSED BETWEEN CML HPCs BEFORE AND AFTER IN VITRO CULTURE WITHOUT IM**

| Gene Symbol | Gene name | Fold change | p-Value | FDR |
| --- | --- | --- | --- | --- |
| GYPA | glycophorin A (MNS blood group) | **13.25** | 0.000454 | 0.008921 |
| HBA2 | hemoglobin, alpha 2 | **12.39** | 0.000296 | 0.00678 |
| CYP1B1 | cytochrome P450, family 1, subfamily B, polypeptide 1 | **11.29** | 0.000828 | 0.012972 |
| PLK1 | polo-like kinase 1 | **10.43** | 0.00001 | 0.001091 |
| PSAT1 | phosphoserine aminotransferase 1 | **7.92** | 0.000043 | 0.002338 |
| KIF20A | kinesin family member 20ª | **7.41** | 0.000001 | 0.00046 |
| DLGAP5 | discs, large (Drosophila) homolog-associated protein 5 | **6.75** | 0.000006 | 0.0009 |
| CDK1 | cyclin-dependent kinase 1 | **6.33** | 9.33E-07 | 0.000431 |
| HMMR | hyaluronan-mediated motility receptor (RHAMM) | **6.24** | 0.000002 | 0.000582 |
| PBK | PDZ binding kinase | **5.84** | 0.000002 | 0.000576 |
| CD40LG | CD40 ligand | -8.1 | 0.00004 | 0.002259 |
| NR4A3 | nuclear receptor subfamily 4, group A, member 3 | **-10.27** | 0.002228 | 0.023754 |
| CXCL8 | chemokine (C-X-C motif) ligand 8 | **-10.92** | 0.000023 | 0.001653 |
| AREG | Amphiregulin | **-11.49** | 0.011381 | 0.072177 |
| ARRDC3 | arrestin domain containing 3 | **-14.68** | 0.000006 | 0.000922 |
| DUSP1 | dual specificity phosphatase 1 | **-18.47** | 0.000001 | 0.000455 |
| NR4A2 | nuclear receptor subfamily 4, group A, member 2 | **-28.35** | 0.000134 | 0.004402 |
| FOS | FBJ murine osteosarcoma viral oncogene homolog | **-29.67** | 2.21E-07 | 0.000189 |
| FOSB | FBJ murine osteosarcoma viral oncogene homolog B | **-31.4** | 7.45E-07 | 0.000364 |
| JUN | jun proto-oncogene | **-39.23** | 0.000005 | 0.000807 |

Top ten genes upregulated and downregulated between CML HPCs cultured without IM for 48 hrs vs HPCs at time 0 (before culture), according to the analysis performed with Affymetrix transcriptome analysis console. Fold Change > 2.0; p <0.05

**TABLE S6**

**TOP TEN GENES DIFFERENTIALLY EXPRESSED BETWEEN CML HPCs BEFORE AND AFTER IN VITRO CULTURE WITH IM**

| Gene Symbol | Gene name | Fold change | p-Value | FDR |
| --- | --- | --- | --- | --- |
| CYP1B1 | cytochrome P450, family 1, subfamily B, polypeptide 1 | **17.87** | 0.000142 | 0.007406 |
| GYPA | glycophorin A (MNS blood group) | **13.79** | 0.000183 | 0.008253 |
| HBA2 | hemoglobin, alpha 2 | **12.02** | 0.00016 | 0.007808 |
| PLK1 | polo-like kinase 1 | **7.42** | 0.000023 | 0.003116 |
| HBG1 | phosphoserine aminotransferase 1 | **7.39** | 0.009897 | 0.084269 |
| GYPB | glycophorin B (MNS blood group) | **6.22** | 0.005267 | 0.057451 |
| KIF20A | kinesin family member 20A | **6.17** | 0.000007 | 0.001821 |
| CDK1 | cyclin-dependent kinase 1 | **6.12** | 0.000016 | 0.002696 |
| SLC7A11 | solute carrier family 7 (anionic amino acid transporter light chain, xc- system), member 11 | **5.62** | 0.000896 | 0.020311 |
| DLGAP5 | discs, large (Drosophila) homolog-associated protein 5 | **5.54** | 0.000027 | 0.003283 |
| NFKBIZ | nuclear factor of kappa light polypeptide gene enhancer in B-cells inhibitor, zeta | **-7.71** | 0.000051 | 0.004559 |
| NR4A3 | nuclear receptor subfamily 4, group A, member 3 | **-7.71** | 0.005117 | 0.056423 |
| NFKBIA | nuclear factor of kappa light polypeptide gene enhancer in B-cells inhibitor, alpha | **-8.54** | 3.23E-07 | 0.000588 |
| AREG | amphiregulin | **-10.64** | 0.019226 | 0.127539 |
| ARRDC3 | arrestin domain containing 3 | **-11.47** | 0.000005 | 0.00155 |
| NR4A2 | nuclear receptor subfamily 4, group A, member 2 | **-20.32** | 0.000304 | 0.011273 |
| DUSP1 | dual specificity phosphatase 1 | **-21.31** | 0.000003 | 0.001166 |
| FOS | FBJ murine osteosarcoma viral oncogene homolog | **-22.95** | 7.31E-07 | 0.000787 |
| FOSB | FBJ murine osteosarcoma viral oncogene homolog B | **-32.17** | 0.000001 | 0.000808 |
| JUN | jun proto-oncogene | **-37.32** | 0.000025 | 0.003227 |

Top ten genes upregulated and downregulated between CML HPCs cultured with IM for 48 hrs vs HPCs at time 0 (before culture), according to the analysis performed with Affymetrix transcriptome analysis console. Fold Change > 2.0; p <0.05.

**FIGURE S1**


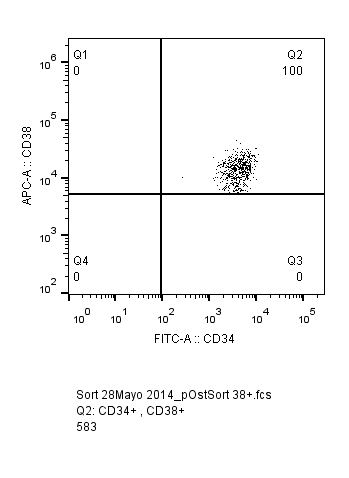

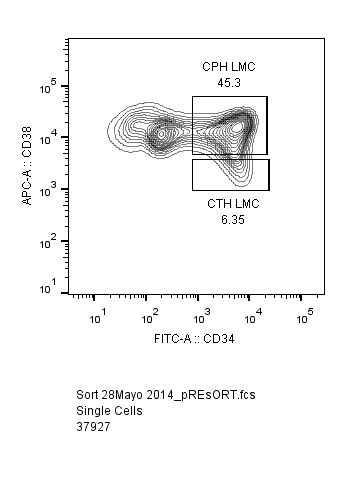


CD34

CD38

CD38

CD34

CD38

CD34


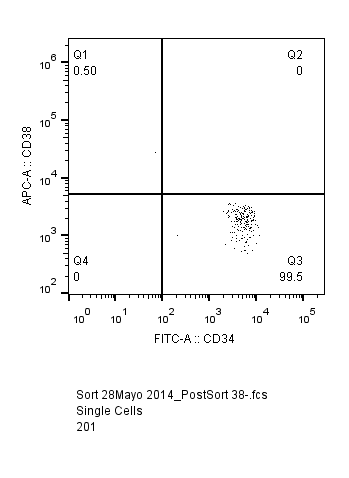


**Purification of HSCs and HPCs**. FACS plots showing the strategy followed for the purification of cell fractions enriched for HSCs (CD34^+^ CD38^-^ Lin^-^ cells; bottom panel on the right) and HPCs (CD34^+^ CD38^+^ Lin^-^ cells; top panel on the right) obtained from CML bone marrow. The same strategy was followed for obtaining both cell populations from normal bone marrow.

**FIGURE S2**

**Effect of IM on the proliferation of HPCs derived from normal (NBM) and CML bone marrow**. Results correspond to the percentage of the total nucleated cell number after 48 hrs of culture in Serum-Free Expansion Media supplemented with a mixture of recombinant cytokines (SCF, TPO, FL, IL-6, IL-3, GM-CSF, G-CSF, and EPO) in the absence (C) or in the presence (IM) of 2.5 µM IM. Total cell number before culture corresponded to 100%.
